# Natural essential oils as a new therapeutic tool in colorectal cancer

**DOI:** 10.1186/s12935-022-02806-5

**Published:** 2022-12-13

**Authors:** Stefania Garzoli, Pedro Alarcón-Zapata, Gulnaz Seitimova, Barbara Alarcón-Zapata, Miquel Martorell, Farukh Sharopov, Patrick Valere Tsouh Fokou, Darline Dize, Lauve Rachel Tchokouaha Yamthe, Francisco Les, Guillermo Cásedas, Víctor López, Marcello Iriti, Javad Sharifi Rad, Eda Sönmez Gürer, Daniela Calina, Raffaele Pezzani, Sara Vitalini

**Affiliations:** 1 Department of Drug Chemistry and Technologies, University “Sapienza” of Rome, P.Le Aldo Moro 5, 00185 Rome, Italy; 2 Clinical Biochemistry and Immunology Department, Faculty of Pharmacy, University of Concepción, Concepción, VIII – Bio Bio Region Chile; 3 Facultad de Ciencias de La Salud, Universidad San Sebastián, Lientur 1457, 4080871 Concepción, Chile; 4 Faculty of Chemistry and Chemical Technology, Al-Farabi Kazakh National University, Almaty, Kazakhstan; 5 Department of Nutrition and Dietetics, Faculty of Pharmacy, and Centre for Healthy Living, University of Concepción, 4070386 Concepción, Chile; 6 Research Institution “Chinese-Tajik Innovation Center for Natural Products”, National Academy of Sciences of the Republic of Tajikistan, Ayni 299/2, Dushanbe, 734063 Tajikistan; 7 Department of Biochemistry, Faculty of Science, University of Bamenda, Bambili, 39 Cameroon; 8 Antimicrobial and Biocontrol Agents Unit, Department of Biochemistry, Faculty of Science, University of Yaounde 1, Ngoa Ekelle, Yaounde, 812 Cameroon; 9 Institute for Medical Research and Medicinal Plants Studies, Yaoundé, 13033 Cameroon; 10 Department of Pharmacy, Faculty of Health Sciences, Universidad San Jorge, 50830 Villanueva de Gállego (Saragossa), Spain; 11 Instituto Agroalimentario de Aragón-IA2 (CITA-Universidad de Zaragoza), 50059 Saragossa, Spain; 12 Department of Biomedical, Surgical and Dental Sciences, Università Degli Studi di Milano, Via G. Pascal 36, 20133 Milan, Italy; 13 Facultad de Medicina, Universidad del Azuay, Cuenca, Ecuador; 14 Faculty of Pharmacy, Department of Pharmacognosy, Sivas Cumhuriyet University, Sivas, Turkey; 15 Department of Clinical Pharmacy, University of Medicine and Pharmacy of Craiova, 200349 Craiova, Romania; 16 Phytotherapy Lab (PhT-Lab), Endocrinology Unit, Department of Medicine (DIMED), University of Padova, Via Ospedale 105, 35128 Padua, Italy; 17 AIROB, Associazione Italiana Per la Ricerca Oncologica Di Base, Padua, Italy; 18 Department of Agricultural and Environmental Sciences, Università Degli Studi di Milano, Via G. Celoria 2, 20133 Milan, Italy

**Keywords:** Colorectal cancer, Natural essential oils, Anticancer properties, Cytotoxicity, Apoptosis, Anticancer therapy

## Abstract

Colorectal cancer (CRC) is the third most revalent type of cancer in the world and the second most common cause of cancer death (about 1 million per year). Historically, natural compounds and their structural analogues have contributed to the development of new drugs useful in the treatment of various diseases, including cancer. Essential oils are natural odorous products made up of a complex mixture of low molecular weight compounds with recognized biological and pharmacological properties investigated also for the prevention and treatment of cancer. The aim of this paper is to highlight the possible role of essential oils in CRC, their composition and the preclinical studies involving them. It has been reviewed the preclinical pharmacological studies to determine the experimental models used and the anticancer potential mechanisms of action of natural essential oils in CRC. Searches were performed in the following databases PubMed/Medline, Web of science, TRIP database, Scopus, Google Scholar using appropriate MeSH terms. The results of analyzed studies showed that EOs exhibited a wide range of bioactive effects like cytotoxicity, antiproliferative, and antimetastatic effects on cancer cells through various mechanisms of action. This updated review provides a better quality of scientific evidence for the efficacy of EOs as chemotherapeutic/chemopreventive agents in CRC. Future translational clinical studies are needed to establish the effective dose in humans as well as the most suitable route of administration for maximum bioavailability and efficacy. Given the positive anticancer results obtained from preclinical pharmacological studies, EOs can be considered efficient complementary therapies in chemotherapy in CRC.

**Supplementary Information:**

The online version contains supplementary material available at 10.1186/s12935-022-02806-5.

## Introduction

Colorectal cancer (CRC) is a devastating disease with a high incidence and mortality rate, accounting for more than 10% of all cancer death in 2020, being the third most common cancer in men and the second in women [1, 2]. According to the International Agency for Research on Cancer, in 2018, the global cancer burden is estimated to have risen to 18.1 million new cases and 9.6 million deaths. CRC is the second largest cancer worldwide with 881,000 deaths in 2018 [3–5]. Studies show that approximately 90% of colorectal cancer cases occur in people over the age of 50 and the number of patients diagnosed with CRC exceeds that of patients diagnosed with lung cancer. CRC has a high rate of treatment success when it is detected in its early stages [6]. Thus the 5-year survival rate is over 90% in early-diagnosed colorectal cancer, but only 40% of tumors are found at a localized stage and approximately 56% of colorectal cancer patients die from the tumor. In stage IV, survival is low, only 14–16% at 5 years [7]. CRC is a type of cancer that affects colon or rectum cells and normally starts on the inner lining of these tissues, where the pathologist can find the so-called polyps [8]. Even if polyps are not strictly malignant, with time they can sometimes enlarge, grow and spread originating cancer. The most common form of CRC is adenocarcinoma and this review analyzed only this kind of tumor, not considering less frequent forms of cancer affecting the colon and rectal tissues, such as carcinoid tumors, gastrointestinal stromal tumors (GISTs), lymphomas, and sarcomas [9, 10]. In general, CRC does not cause symptoms at the initial stages, even if small blood loss, fatigue, lack of appetite, anemia, weight loss, stubborn constipation, alternating with diarrhea can be present [11]. The diagnosis of CRC is based on an accurate anamnesis, followed by blood tests with the research of carcinoembryonic tumor marker (CEA), digital rectal examination and colonoscopy with biopsies. In addition, ultrasound, computed tomography (CT) and magnetic resonance imaging can be used to assess the extent of the tumor itself and the presence or absence of distant metastases [12]. More recently, clinicians are beginning to use the results of the molecular profile of CRC from biopsy, as it can serve to better define the prognosis and therapy of this neoplasia [13]. The therapeutic strategy is essentially based on surgery, which can be assisted by chemotherapy and radiotherapy, alone or combined, adjuvant or neoadjuvant. Moreover, targeted therapy and immunotherapy are two recent therapeutic tools for the management of aggressive, advanced or metastatic CRC [14, 15]. Nonetheless, these strategies have numerous adverse side effects, and as a result, new adjuvant therapies in the treatment of cancer have been sought, the naturally bioactive compounds being known as potential anticancer adjuvant and complementary agents [16–19]. The study of natural products has always guided the field of applied pharmacology [20]. They have played a key role in drug discovery, especially for cancer and infectious diseases [21, 22]. In the area of cancer, since the 1940s, more than 50% of the active molecules are unaltered or derivative natural products of different origins (plant, animal and microbial) [16, 23]. Among these, some examples are paclitaxel (Taxol^®^), vincristine (Oncovin^®^), vinorelbine (Navelbine^®^), teniposide (Vumon^®^) and various water-soluble analogues of camptothecin (e.g. Hycamtin^®^) [24–26]. After a decline in the pharmaceutical industry's search for natural products from the 1990s onwards due to technical barriers to screening, isolation, characterization and optimization, in recent years, technological and scientific development has revitalized the interest in them [27, 28]. According to Newman & Cragg [26], natural products still offer the best potential for discovering new compounds that can lead to effective agents in a variety of human diseases. Essential oils, complex mixtures of volatile organic compounds extracted from plants by steam distillation, dry distillation or a suitable mechanical process without heating, possess biological and pharmaceutical properties including anticancer activity [29]. Various types of malignancies are reported to be lowered after treatment with essential oils [30]. The current status of knowledge regarding their potential in the treatment strategies of CRC, the second deadliest (about 1 million per year) and third most commonly diagnosed cancer in the world (about 2 million cases in 2020) [1], is covered in this review.

## Essential oils: a brief overview

### Traditional uses

Humans use medicinal plants for disease treatment for a long time [27, 31–33]. Such a traditional method possesses more than thousands of years of history, as noted by ancient Persian, Indian, Chinese, Arabic, and Greek manuscripts [20, 34]. Among natural phytochemicals, EOs have attracted human attention due to their pleasant aroma [35]. They have been ethnotraditionally used for the treatment and prevention of various diseases by different human cultures [36]. The application of EOs against neoplasia is a very promising field [37]. In 2005, Warnke and coauthors reported that the application of tea tree and eucalyptus oils has reduced tumor smell and inflammation in cancer patients [38]. *Rosa x damascena* has a long history of use in traditional medicine: its EO could increase cell proliferation on SW742 when higher concentrations were used, i.e. 10 μg/mL with 48 h of incubation time [39]. Moreover, the same work showed that similar effects were perceived in human normal fibroblasts, thus inducing the authors to suggest that the EO of *Rosa x damascene* could stimulate cell growth. EOs extracted from 6 sand-dune plants of Portugal region (*Seseli tortuosum* L., *Otanthus maritimus* (L.) Hoffmanns. & Link, *Eryngium maritimum* L., *Crithmum maritimum* L., *Artemisia campestris* subsp. *maritima* (DC.) Arcang., *Juniperus phoenicea* var. *turbinate* (Guss.) Parl.,) reported promising cytotoxic properties [40]. In 1997, Gould has postulated that the naturally occurring monoterpenes are a potential new class of potential anticancer agents [41]. For example, D-limonene showed anticancer activity against many rodent solid tumor types by carcinogen detoxification and inhibition of the posttranslational isoprenylation of growth-controlling small G proteins (p21ras) [42].

### Chemical composition

Many plants produce volatile terpene substances in their vital processes. In addition to terpene hydrocarbons, some volatile molecules have various oxygen or sulphur-containing functional group such as hydroxyl, carbonyl, carboxyl, thiol and others [43, 44]. The mixture of these compounds commonly called EOs contains more than twenty constituents at varying concentrations with two–three major components [45]. EOs are characterized by complex chemistry due to a set of aromatic substances known as secondary low molecular weight metabolites [46]. These molecules belong to several classes of compounds predominantly represented by monoterpenes [47], sesquiterpenes [48] and their derivatives. EOs are obtained by hydro- or steam distillation starting from different parts of the plant such as leaves, flowers, and stems[35]. Precisely because of their rich and varied chemical composition, accurate characterization by gas chromatography and mass spectrometry is essential.

## Anticancer mechanisms of EOs: molecular evidence from preclinical studies

**Fig. 1:**
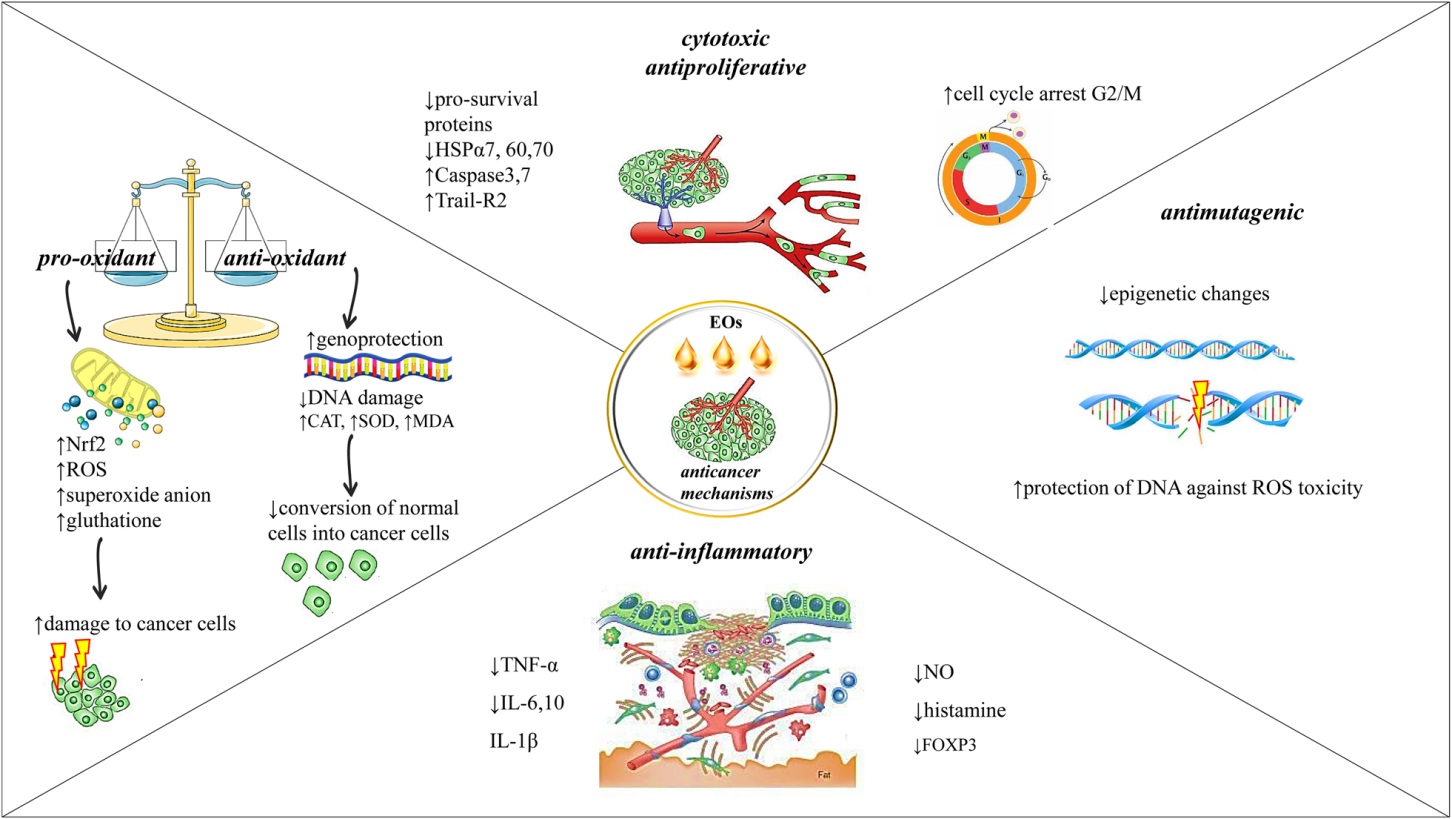
Diagram with the most representative anticancer molecular mechanism of natural EOs. Legend: ↑increase, ↓decrease, Nuclear factor erythroid 2-related factor 2 (Nrf2), Reactive oxygen species (ROS), Deoxyribonucleic Acid (DNA), Heat shock proteins (Hsps), Interleukin (IL), Tumor necrosis factor (TNF), Forkhead box P3 (FOXP3)

Various EOs have been studied in many types of experimental models in the search for new treatments for colon cancer with very promising results [16, 20] (Additional file 1). EOs exhibit a wide range of bioactive effects like cytotoxicity, antiproliferative, and antimetastatic effects on cancer cells through various mechanisms of action [49]. It has been demonstrated that EOs possessed, for the most part, a prodigious activity directed against cancer cells [45]. In the case of CRC, the effect of EOs has been analyzed in vitro studies on human colon cancer cell lines such as HT-29, Caco-2, SW480, and HCT-116, among others. HCT-116 cells have been the most studied since they are classified as a cell line model to study the molecular mechanisms involved in tumor metastasis [50]. The EOs' bioactive compounds against colorectal cancer models are summarized in Table 1 and Fig. 1.

**Table 1:** Chemical composition of EOs and the correlation with different CRC experimental models

Plant species	Family of plants	Tested CRC cell lines	Concentration	Potential mechanism	Main chemical compounds	Refs.
*Lavandula* *stoechas ssp. stoechas*	Lamiaceae Labiatae	COL-2	IC_50_ = 9.8 μg/mL	Not available	pulegone, hexahydrothymol, menthone	[51]
*Annona Cheirmola* *Annon squamosa* *Annona Abdel Razik*	Annonaceae	HCT116	IC_50_ = 2.1 μg/mL IC_50_ = 0.7 μg/mL IC_50_ = 0.7 μg/mL	Not available	α-pinene, β-pinene, α-copaene, 2-carene isocaryophyllene, caryophyllene	[52]
*Cymbopogon flexuosus*	Poaceae	HT-29 HCT-15 SW-620 502,713	IC_50_ = 42.4 μg/mL IC_50_ = 60.2 μg/mL IC_50_ = 28.1 μg/mL IC_50_ = 4.2 μg/mL	Not available	not available	[53]
*Illicium verum*	Asteraceae Compositae	HCT-116 HT-29 CCD-18co	IC_50_ = 50.34 μg/mL IC_50_ = 100 μg/mL IC_50_ = 200 μg/mL	↑Apoptosis ↓ Metastasis	tans-anethole elaidic acid palmitic acid	[54]
*Commiphora molmol*	Burseraceae	HCT-116	IC_50_ = 19.71 μg/mL	Not available	2-acetoxy-furano-diene furanoeudesma-1,3-diene furanoeudesma-1,4-dien-6-one isofuranogermacrene	[55]
*Capparis spinosa L*	Burseraceae	HT-29	Not available	↓Proliferation, ↓NF-kB no apoptosis in HT-29 cells ↑Cell cycle arrest G2/M phase	methyl isothiocyanate	[56]
*L. hybrid Re* *L. latifolia Medikus* *L. vera D.C*	Lamiaceae Labiatae	Caco-2	IC_50_ = 0.9132 mg/mL IC_50_ = 0.7798 mg/mL IC_50_ = 1.224 mg/mL IC_50_ = 1.631 mg/mL	↑ROS/RNS, ↓Akt ↓mTOR ↓MAPK, ↓NF-κB	linalool linalyl acetate 1,8-cineole	[57]
*Cinnamomum glanduliferum* Bark	Lamiaceae	HCT-116	IC_50_ = 9.1 μg/mL	Not available	eucalyptol, terpinen-4-ol α -terpineol	[58]
*Achillea* *fragrantissima*	Asteraceae	SW48 HCT116	IC_50_ = 110.1 μg/mL IC_50_ = 134.6 μg/mL	Not available	*Artemisia* ketone camphor, α-bisabolol	[59]
*Grapefruit*	Rutaceae	HCT116	Not available	Dose-dependent antiproliferative activity	nerylisovalerate, 1,8-cineole neryl-2-methyl-butanoate chamazulene, linalool, camphor germacrene D, nerol linalyl propionate	[60]
*Artemisia dubia Wall*	Asteraceae		IC_50_ = 31.25 μg/mL	Not available	limonene, linalyl acetate γ-terpinene, linalool β-pinene, bergapten	[61]
*Ocimum viride*	Lamiaceae Labiatae	HT-29 502,713 SW-620	IC_50_ = 0.034 μL/mL	↑DNA damage ↑Cells death ↑Apoptotis	thymol, γ -terpinene p-cymene	[62]
*Cinnamomum stenophyllum* (Meisn.) Vattimo-Gil	Lauraceae	HCT116	IC_50_ = 9.95 μg/mL	Not available	eugenol, safrol, benzyl benzoate, 1,8-cineole, camphor	[63]
*Citrus aurantifolia* (Christm.) Swingle	Rutaceae	NIH3T3 SW-480	IC_50_ = 6.25 μg/mL	↑DNA fragmentation ↑caspase-3 ↑Bax/Bcl2	D-Limonene, D-Dihydrocarvone α -Terpineol	[64]
*Citrus limettioides*	Rutaceae	SW480	IC_50_ = 50 μg/mL	↑Apoptosis	d-Limonene triacontane α-Bisabolene α-Farnesene (R)-( +)-Citronellol	[65]
*Heracleum pastinacifoliu* *Heracleum persicum* *Heracleum rechingeri* *Heracleum transcaucasicum*	Apiaceae	LS180	IC_50_ = 1.4 mg/mL	Not available	myristicin (E)-anethole hexyl butanoate elemicin	[66]
*Comptonia peregrina* L	Myricaceae	DLD-1	IC_50_ = 47 μg/mL	Not available	β -caryophyllene, α-humulene, β -myrcene	[67]
*Cotula cinerea* (Delile)	Asteraceae	HCT116	IC_50_ = 86.7 μg/mL IC_90_ = 122.3 µg/mL	Not available	trans-thujone santolina triene, α- pinene, sabinene, 1,8-cineole	[68]
*Leonotis nepetifolia*	Lamiaceae Labiatae		IC_50_ = 16.78 mg/mL	Not available	germacrene D, α-humulene, 3-octanone, (E)-ocimene, (Z)-ocimene, linalool, β -caryophyllene, 1-octen-3-ol	[69]
*Eryngium campestre Eryngium amethystinum*	Apiaceae		IC_50_ = 1.65 μg/mL IC_50_ = 1.64 μg/mL	Not available	germacrene D, spathulenol, alloaromadendrene, ledol, γ-cadinene, β -Elemene	[70]
*Tagetes erecta* L	Asteraceae	HT29	IC_50_ = 6.94 μg/mL	Not available	limonene (10.4%), dihydrotagetone (11.8%), terpinolene (18.1%), (E)-ocimenone (13.0%)	[71]
*Tetradenia riparia (Hochst.)*	Lamiaceae Labiatae		IC_50_ = 77.47 μg/mL		fenchone (6.1%), dronabinol (11.0%), aromadendrene oxide (14.7%) (E,E)–farnesol (15.0%)	
*Bidens sulphurea (Cav.)*	Asteraceae		IC_50_ = 268.8 μg/mL		(E)-caryophyllene(10.5%) germacrene D (35.0%) 2,6-di-tert-butyl-4-methylphenol (43.0%)	
*Foeniculum vulgare Mill.,*	Apiaceae		Not available		limonene (21.3%) (E)-anethole (70.2%)	
*Piper betel L*	Piperaceae	RCM-1	IC_50_ = 500 μg/mL	Duct formation after treated with EO	chavicol, chavibetol, cineol, eugenol	[72]
*Cymbopogon nadus L*	Poaceae				octyl acetate (54.9–60.2%) octyl butyrate (10.1–13.4%)	
*Syzygium aromaticum* L	Myrtaceae				eugenol, eugenyl acetate, β-caryophyllene, α-humulene	
*Alpinia galanga* L	Zingiberaceae				1,8-cineol, alpha-pinene, eugenol, camphor, methyl cinnamate	
*Psidium guajava* L	Myrtaceae				β-caryophyllene, cineol	
*Ocimum americanum* L	Lamiaceae Labiatae				limonene, 1,8-cineol, δ-cadinene, α-pinene α-terpineol	
*Ocimum tenuiflorum* L					camphor, cineol, eugenol, limonene, rosmarinic acid	
*Citrus hystrix* DC	Rutaceae				β-pinene, limonene, caryophyllene, sabinene, citronellol, 1,8-cineol	
*Cymbopogon* *citratus* (DC) Stapf	Poaceae				citral, myrcene, geraniol, nerol, farnesol, citronellol	
*Boesenbergia* *rotunda* (L.) Mansf	Zingiberaceae				camphene, eucalyptol, ocimen, camphor, geraniol	
*Citrus aurantifolia* (Christm. et Panz.) Swings	Rutaceae				D-limonene, pinene, camphene, bergapten	
*Ocimum basilicum* L	Lamiaceae Labiatae				estragole, linalool, 1,8-cineole	
*Curcuma* *longa* L	Zingiberaceae				turmerone, aromatic (ar-) turmerone	
*Rosa damascena*	Rosaceae	SW742	IC_50_ = 10 μg/mL	↓Cell proliferation	not available	[39]
*Pistacia atlantica*	Anacardiaceae	Caco-2 HCT116	IC_50_ = 62.85 μg/mL IC_50_ = 34.97 μg/mL	Not available	α-pinene, sabinene, limonene, terpinene-4-ol, β-pinene	[73]
*Phoebe bournei* (Hemsl.)	Laureaceae	SW480	IC_50_ = 41.3 l μg/mL	Not available	α-copaene, α-muurolene, δ-cadinene, 1 s-calamenene	[74]
*Ammodaucus* *leucotrichus* Cosson & Durieu	Apiaceae	HCT116	IC_50_ = 41.3 l μg/mL	Not available	perillaldehyde, D- limonene α-pinene	[75]
*Inula graveolens* (Linnaeus) Desf	Linnaeus	HT29	IC_50_ = 24.6 μg/mL	Not available	bornyl acetate, corneol caryophyllene oxide δ-cadinol, camphene	[76]
*Ocimum viride*	Lamiaceae Labiatae	COLO 205	IC_50_ = 0.070, 0.058, 0.033 μg/mL at 24, 48, and 72 h, respectively	↑DNA damage ↑mitochondrial membrane permeability ↑apoptosis	thymol, α-pinene geranyl acetate β-caryophyllene oxide	[77]
*Artemisia indica* Wild	Asteraceae	Caco-2	IC_50_ = 19.5 μg/mL	Not available	*Artemisia* ketone, germacrene B borneol cis-chrysanthenyl acetate	[78]
*Pogostemon cablin*	Lamiaceae Labiatae	HCT116 SW480	Not available	↑p21 ↓Cyclin D1 ↓CDK4	not available	[79]
*Eugenia uniflora*	Myrtaceae	HCT-116	IC_50_ = 16.26 μg/mL IC_50_ = 9.28 μg/mL	Not available	curzerene, selina-1,3,7(11)-trien-2- one, selina-1,3,7(11)-trien-2-one epoxide, germacrene B, caryophyllene oxide, (E)-caryophyllene	[80]
*Origanum onites* L	Origanum	HT-29	IC_50_ = 0.35 μg/mL	Not available	terp-1-in-4-ol, sabinene hydrate, γ-terpinene, p-cymene α-terpineol	[81]
*Stachys viticina* Boiss	Lamiaceae Labiatae	Colo-205	Not available	Not available	endo-borneol, eucalyptol epizonarene	[82]
*Moringa oleifera*	Moringaceae	Caco-2	↑Cytotoxicity	↑Morphological alterations ↑Cell blebbing and vacuolation ↑Autophagy ↑Cancer cell death	not available	[83]
*Citrus bergamia Risso et Poiteau*	Rutaceae	Human and rat isolated CRC cells	Not available	EOs inhibited neuronally-mediated contractions in the rat and human CRC	(R)-( +)-limonene linalyl acetate linalool	[84]
*Melissa officinalis*	Lamiaceae Labiatae	HT-29 T84	IC_50_ = 346 μg/mL IC_50_ = 120 μg/mL	↑Cell cycle arrest ↑Apoptosis	not available	[85]
*Mesua ferrea*	Calophyllaceae	HCT 116 IM1215	IC_50_ = 17.38 μg/mL IC_50_ = 18.86 μg/mL	↑Morphological and biochemical changes in HCT 116	isoledene, elemene	[86]
*Origanum majorana*	Lamiaceae Labiatae	HT-29	Not available	↑Autophagy ↑Apoptotis ↑p38, ↑MAPK	terpinen-4-ol, alpha-terpinol α-pinene, camphene, p-cymol β-caryophyllene, bicyclogermacrene, neophytadiene	[87]
*Thymus alternans*		HCT-15 HCT116	IC_50_ = 5–8 l μg/mL	Not available	(E)-nerolidol, (E)-β-Ocimene geranial	[88]
*Mentha citrata*		HCT116	IC_50_ = 80.6 μg/mL IC_90_ = 119.1 μg/mL	Not available	linalool. linalyl acetate 1,8-cineole, a-terpineol	[89]
*Teucrium* *alopecurus*			Not available	↑Apoptosis ↓Cells survival ↓Proliferation ↓Invasion ↓Angiogenesis ↓Metastasis	( +)-epi-bicyclo sesquiphellandrene α-bisabolol, Ƭ-muurolol α-cadinol, β-phellandrene d-limonene	[90]
*Ocimum basilicum,* *Mentha spicata,* *Pimpinella anisum,* *Fortunella margarita*	Lamiaceae Apiaceae Rutaceae	Caco_2_	Sweet basil IC_50_ = 0.071 mg/mL Kumquat IC_50_ = 0.1 mg/mL Spearmint IC_50_ = 0.162 mg/mL Anise IC_50_ = 0.25 mg/mL	Not available	carvone in spearmint methyl chavicol in sweet basil trans-anethole in anise limonene in kumquat	[91]
*Pinus roxburghii*	Pinaceae	HCT116	IC_50_ = 25.0 μg/mL	↑Apoptosis	α-pinene caryophyllene oxide 3-carene β-pinene	[92]
*Artemisia santonicum*	Asteraceae	HCT116	Not available	↓Pro-inflammatory factors ↓Cell growth ↓Cancer cells survival	camphor, 1,8-cineole α-thujone borneol, β-thujone	[93]
*Smyrnium olusatrum* L	Apiaceae	HCT116	IC_50_ = 10.71 μg/mL	↑DNA fragmentation ↑Phosphatidylserine ↑Caspase-3	isofuranodiene germacrone furano-4(15)-eudesmen-1-one furanoeremophil-1-one 1β-acetoxyfuranoeudesm-4(15)-ene	[94]
*Zataria multiflora* Boiss	Lamiaceae Labiatae	HCT116 SW48	Not available	↓Cell proliferation ↑Apoptosis	not available	[95]
*Zedoary Turmeric*	Zingiberaceae	HCT116	IC_50_ = 101 μg/mL	↓Growth of cancer cells ↑Senescence ↑Apoptosis	not available	[96]
*Artemisia campestri*	Asteraceae	HT-29	Not available	Not available	*A.Campestris*: β-pinene, limonene,, germacrene-D, γ-terpinene, β-myrcene, α-pinene, (Z)-β-ocimene (E)-β-ocimene	[97]
*Croton lechleri*	Cynomoriaceae	LoVo	IC_50_ = 74.95 μg/mL	↑Change in fatty acid composition	sesquicineole, α-calacorene, 1,10-di-epi-cubenol,, β-calacorene, epicedrol	[98]
*Allium Roseum* L	Alliaceae	HT-29 Caco-2	IC_50_ = 4.64 μg/mL IC_50_ = 8.22 μg/mL	Not available	methyl methanethiosulfinate, 3-vinyl.1,2-dithiacyclohex-5-ene diallyl trisulfide	[99]
*Chrysanthemum coronarium* L	Asteraceae	Caco-2	IC_50_ = 43.0 μg/mL	Not available	not available	[100]
*Beilschmiedia erythrophloia*	Lauraceae	HT-29	IC_50_ = 18.9 μg/mL	Not available	β-caryophyllene, α-humulene terpinen-4-ol, cis-β-ocimene, sabinene, limonene	[101]
*Machilus mushaensis*	Lauraceae		IC_50_ = 3.8 μg/mL	Not available	n-decanal, α-cadinol	[102]
*Porcelia macrocarpa*	Annonaceae		IC_50_ = 50.8 μg/mL	Not available	germacrene D, bicyclogermacrene	[103]
*Neolitsea variabillima*	Lauraceae		IC_50_ = 16.8 μg/mL	Not available	β-ocimene, α-cadinol, terpinen-4-ol, τ-cadinol, β-caryophyllene, sabinene	[104]
*Diospyros discolor*	Ebonaceae		IC_50_ = 10.6 μg/mL	Not available	(2Z,6E)-farnesol, α-cadinol, (E)-nerolidol, Ƭ-cadinol, Ƭ-muurolol, α-humulene, β-caryophyllene	[105]
*Machilus thunbergii*	Lauraceae		IC_50_ = 3.8 μg/mL	Not available	n-decana, β-caryophyllene α-humulene, β-eudesmol	[106]
*Salvia libanotica*	Lamiaceae Labiatae	HCT116 p53 + / + HCT116 p53-/-	Not available	↑Apoptosis ↑Caspase-3 in p53 + / + cancer cells but not p53-/- cells	not available	[107]
*Origanum vulgare*	Lamiaceae Labiatae	HT-29	Not available	Not available	4-terpineol, thymol, γ-terpinene, carvacrol	[108]
*Athanasia brownii*	Asteraceae	HCT116	IC_50_ = 29.5 μg/mL	Not available	selin-11-en-4a-ol, caryophyllene oxide humulene epoxide II (E)-nerolidol	[109]
*Afrostyrax lepidophyllus,* *Scorodophloeus zenkeri*	Huaceae, Fabaceae		IC_50_ = 12.4 μg/mL IC_50_ = 8.5 μg/mL	Not available	2,4,5,7-tetrathiaoctane	[110]
*Salvia* *officinalis*	Lamiaceae Labiatae	HT-29 Caco-2 HCT116	Not available	↑Morphological changes	α-thujone 1,8-cineole, camphor	[111]
*Hedychium spicatum*	Zingiberaceae	LD-1 SW620	IC_50_ = 26.75–94.35 mg/mL	Not available	1,8-cineol, hedycaryol, β-eudesmol, Ƭ-eudesmol, cubenol, α-cadinol	[112]
*Allium sativum*	Allium	HT-29 c	Not available	↑Apoptotis	not available	[113]
*Moringa oleifera*	Moringaceae	Caco-2	Toxicity% = 49.7%	↓Cell viability	not available	[83]
*Myristica fragrans*	Myristicaceae	Caco-2	Not available	Not available	myristicin, sabinene, α-pinene β-pinene, β-Phellandrene safrole, terpinen-4-ol	[114]
*Callistemon citrinus*	Myrtaceae	Colo-205	Not available	Not remarkable activity	α-pinene, limonene α-terpineol in leaf oil, 1,8-cineole α-pinene in flower oil	[115]
*Eugenia egensis* *Eugenia flavescens* *Eugenia polystachya* *Eugenia patrisii*	Myrtaceae	HCT-116	IC_50_ = 10.5–216.3 mg/mL	↑Cell membrane disruption	5-hydroxy-cis-calemene (2E,6E)-farnesol, (2E,6Z)-farnesol caryophylla-4(12),8(13)-dien-5-ol-5 -ol E–bisabolene, germacrene D, and ishwarane	[116]
*Aquilaria crassna*	Thymelaeaceae		IC_50_ = 28.0 μg/mL	↑Apoptotis ↑DNA fragmentation ↑mitochondrial damage	β-caryophyllene 1-Phenanthrenecarboxylic acid 2-naphthalene-methanol α-caryophyllene benzenedicarboxylic acid Azulene, naphthalene, cyclodecene	[117]
*Nectandra leucantha*	Lauraceae	HCT	IC_50_ = 194.8 μg/mL	Not available	bicyclogermacrene germacrene A spathulenol, globulol	[118]
*Semenovia suffruticosa*	Apiaceae	HT-29	IC_50_ = 341 μg/mL	Morphological changes	Z-β-ocimene linalool, β-bisabolol	[119]
*Piper aequale*	Piperaceae	HCT-116	IC_50_ = 8.68 μg/mL	Not available	δ-elemene, β-pinene, α-pinene, cubebol, β-atlantol bicyclogermacrene	[120]
*Pistacia lentiscus* var. *chia*	Anacardiaceae	HT-29 Caco-2 CT26	IC_50_ = 0.1752 mg/mL IC_50_ = 0.0368 mg/mL IC_50_ = 0.1335 mg/mL	↓Proliferation of colon cancer cells	α-pinene myrcene	[121]
*Faeniculum* *vulgare*	Apiaceae	HCT-116	Not available	↓DNA damages ↓mitochondrial membrane potential loss	not available	[122]

Symbols: ↑increase, ↓decrease

### Cytotoxic and antiproliferative effects of EOs

The EO from rhizome of *Curcuma purpurascens* BI. demonstrated cytotoxic effects against HT-29 cells (IC_50_ value 4.9 ± 0.4 μg/mL) [123]. In 2009, Sharma and coworkers reported that the lemongrass EO (*Cymbopogon flexuosus* (Nees ex Steud.) W.Watson) showed promising anticancer activity and caused a loss in tumor cell viability by activating the apoptotic process. The IC_50_ values were 4.2 and 4.7 μg/ml for 502,713 (colon) and IMR-32 (neuroblastoma) cell lines, respectively [53].

Blood oranges EO, a variety of orange (*Citrus* × *sinensis*), showed pro-apoptotic and anti-angiogenesis potential on colon cancer cells [124]. Volatile EO isolated from *Artemisia campestris* L. exhibited significant antitumor activity against the HT-29 cells and it is recommended for further research into the chemoprevention and treatment [97, 125].

Thymoquinone (TQ) is a volatile secondary metabolite found in many species including *Nigella sativa* L. (black cumin) and *Monarda fistulosa* L. This monoterpene exhibited anti-proliferative activity against Caco-2, HCT-116, LoVo, DLD-1 and HT-29 cell lines, but not against human intestinal FHs74Int cells [126]. More recently two interesting reviews extensively explored TQ effects in preclinical settings. The first one suggested as TQ could increase the efficacy of chemotherapeutic agents in CRC, in addition to other common cancers (i.e. lung, liver, breast, prostate, etc.), inducing the authors to recommend this combination strategy to fight cancer [127]. Similarly, the other work investigated the combination regimen of TQ and chemotherapy, but also examined the use of nanotechnologies incorporating TQ, encouraging clinicians to test this molecule in clinical trials [128].

Carvacrol-rich EO of *Origanum onites* L. was evaluated in twenty female BALB/c mice xenograft of colon cancer cells (CT26). *Origanum onites* EO was administered orally at a daily dose of 0.370 g/kg of animal body weight for 13 days [81]. The authors reported that colon cancer cells were the most sensitive to *Origanum onites* EO [55, 81]. On the same line, another work obtained an IC_50_ of carvacrol corresponding to 92 µM and 42 µM for HCT-116 and HT-29 cells, respectively [129].

EOs obtained from *Rosa x damascena* were studied in 2D cell models of the RKO cell line (colorectal cancer) and HEK293-T cell line (human embryonic kidney), while for 3D cell models were used only MCF7 cell line (breast cancer). Indeed, in the RKO 2D cell line, the most active EOs were those extracted by *S. tortuosum* and *O. maritimus*, which possessed an IC_50_ of 0.034 and 0.34 μl/mL, respectively. Moreover, Murata and collaborators showed that 1,8-cineole exerted antitumor activity on HCT-116 and RKO cell lines [130]. The authors explored the antiproliferative effect of 1,8-cineole, with an IC_50_ > 10 mM for both cell lines and found induction of apoptosis via activation of the caspase-dependent pathway starting as 25 mM for RKO cell lines.

The EO of *Cinnamomum stenophyllum* (Meisn.) Vattimo leaf has been shown potent cytotoxic effect on HCT-116 cells [131]. This effect on HCT-116 cells has been demonstrated by exposing cells to: a) extracts of 3 *Annona* species (*A. squamosa* L., *A. cherimola* Mill., and the hybrid between them—*Abdel Razek*), b) oil and extracts of *Eugenia uniflora* L., and EO of *Commiphora myrrha* (Nees) Engl., c) EO from *Piper aequale* Vahl, d) EO from *Thymus roegneri* K. Koch, e) EO isolated from the bark of *Cinnamomum glanduliferum* (Wall.) Meisn., f) light phase obtained by molecular distillation of grapefruit [55, 58, 60, 80, 88, 120, 132]. The same cytotoxic effect has been achieved in HT-29 cells by exposing them to *Dittrichia graveolens* (L.) Greuter EO, *Machilus thunbergii* Siebold & Zucc. EO and *Diospyros discolor* Willd. flower. The latter two also exhibited antineoplastic properties on a panel of cancer cells, suggesting a wider effect in different cancer types [105, 106, 133]. On the other hand, the EO of bulbs *Allium roseum* L. presented an antiproliferative dose-dependent effect against two human colon adenocarcinoma cell lines, HT-29 and Caco-2 [99]. Besides, the EO of *Achillea fragrantissima* (Forssk.) Sch.Bip. revealed an IC_50_ of 110.1 and 134.6 µg/mL on human colorectal cancer cells (SW480 and HCT-116) [59].

In a recent study, anticancer effects of *Eryngium campestre* L. and *Eryngium amethystinum* L. were studied, showing IC_50_ values (1.65–5.32 and 1.5–2.99 µg/mL for *E. amethystinum* and *E. campestre*, respectively) comparable or close to those of the chemotherapeutic drug cisplatin [70]. A more selective effect was observed with *Brocchia cinerea* (Delile) Vis. EO, in two human cancer cell lines HCT-116 and liver cancer cell line (HePG2): the results indicated that such EO possessed a significant (66.9%) cell growth inhibition capacity in colon cancer cells [134].

Eucalyptol from the EO of *Cinnamomum glanduliferum* (the main compound) showed great antitumoral potential in HCT-116 cells with IC_50_ of 9.1 μg/ml [58]. Moreover, the molecular mechanisms of *Mesua ferrea* L. oil-gum resin extract on colon cancer cells HCT-116 and LIM1215 were studied. The extract could negatively regulate the expression of multiple pro-survival proteins, such as survivin, xIAP, HSP27, HSP60, and HSP70, and increase the expression of reactive oxygen species (ROS), caspase-3,7 and TRAIL-R2 in HCT-116 [86].

It has been demonstrated that the EO of *Salvia officinalis* L., showed antiproliferative effect based on cell cycle arrest. Through MTT test, at 72 h Caco-2, HT-29, and HCT-116 cells were treated with different concentrations of EOs, exhibiting a dose-dependent cell growth inhibition. Moreover, when analyzing all the possible combinations of the 3 main compounds of the EO, i.e. α-tujona, eucalyptol, and camphor, the same effect was observed. In addition, *S. officinalis* EOs induced cell cycle arrest at the G2/M phase in Caco-2 and HCT-116 cells and the S phase in HT-29 cells. Concomitantly, the treatment with a combination of the three main components increased the percentage of Caco-2 and HCT-116 cells in G0/G1 and HT-29 cells in G2/M. It is worth highlighting that normal colon epithelial cell line FHC was not affected by the same treatment [111]. It also analyzed the effect on cell proliferation of *Melissa officinalis* L. extract on HT-29 and T84 human colon adenocarcinoma cells. The results showed that after 3 and 4 days of treatments there was a growth inhibition of HT-29 and T84 cells with an IC_50_ of 346 and 120 μg/mL, respectively. This antiproliferative effect was associated with a cell cycle arrest in the G2/M phase [85].

Another study suggested that certain EOs might have a chemopreventive and antimetastatic effect. For example, EOs obtained from the fruits of *Illicium verum* Hook. f. decreased cell migration ability of HCT-116 cells in a dose-dependent manner (25, 50, and 90 μg/mL), already at 24 h of treatment [50]. Differently from previous findings, other EOs showed lesser anticancer effects. The EO of *Leonotis nepetifolia* (L.) R.Br. and several isolated compounds (hentriacontane, phyllo palmitate, stigmasteryl glycoside, 6,7-dimethoxy-5,3',4'trihydroxyflavone, apigenin-7-*O*-glucoside, and luteolin-7-*O*-glucoside) showed a low cytotoxic effect on HCT-116 cells [69]. In addition, EOs from different *Eugenia* species (*E. egensis* DC., *E. flavescens* DC., *E. polystachya Rich.*, and *E. patrisii* Vahl) revealed that the most active EO was extracted from *E. polystachya,* at least in HCT-116 cell model. The *E. flavescens* and *E. patrisii* EOs, on the other hand, showed greater toxicity on normal MRC5 cells (human fibroblasts) [116]. Overall, these results do not limit the possibility of improving and innovating the cancer therapy by EOs, rather they should be considered as a stimulus to search for a more successful and reliable therapy against CRC.

### Pro-oxidant and antioxidant effects of EOs

Oxidative stress is one of the causes of cell and DNA damage that can trigger the development of many diseases. [21, 135–137]. The use of a pro-oxidant strategy has been proposed to damage the modified tissues selectively [138]. Therefore, the search for bioactive compounds with antioxidant capacity is a strategy to prevent this problem [139–141]. Numerous studies are showing antioxidant properties using in vitro tests such as DPPH or FRAP, but few exist in cell lines of human colon cancer (i.e. Caco-2, HCT-116, LoVo, DLD-1 and HT-29).

EOs from the bulb of *Allium roseum* L., rich in sulphur compounds as methyl methanethiosulfinate, showed an interesting antiproliferative activity against HT-29 and Caco-2 cells in a dose-dependent manner. It also showed antioxidant activity in FRAP and DPPH assays, and the ability to inhibit the production of superoxide anion in the above-mentioned cell lines [99]. In another study, the treatment of HCT-116 and HT-29 cells and primary fetal colon cells (FHC) with cinnamaldehyde and an ethanolic extract of cinnamon bark (*Cinnamomum cassia* (L.) J.Presl), upregulated cellular protein levels of Nrf2, increased cellular levels of glutathione and protected HCT-116 cells against hydrogen peroxide-induced genotoxicity and arsenic-induced oxidative damage [142].

The antioxidant activity of EOs could protect DNA and tissues from damage caused by oxidative stress and ROS (reactive oxygen species) [143, 144]. A recent study in HT-29 cells showed that certain chemical compounds in EOs such as nerolidol, thymol, geraniol, methyl isoeugenol, eugenol, linalool and a commercial mixture (Agolin) showed antioxidant as well as cytotoxic activity against this cell line [145]. Genoprotection against oxidative DNA damage was also observed for all studied compounds, being thymol (at 12.5 ppm) the most protective compound against oxidative DNA damage. Geraniol (at 125 ppm) also protected cells against DNA damage by methylation. Another study investigated the cytotoxic, genotoxic, and DNA protective effects of carvacrol and thymol in HepG2 and Caco-2 cell lines. Both compounds did not induce DNA chain breaks in any cell line, and in the presence of hydrogen peroxide, they offered significant protection against DNA strand breaks [146].

The effects of fennel EO, *Foeniculum vulgare* Mill., were evaluated against the toxicity induced by an insecticide-triflumuron in HCT116 cells [5]. When cells were pretreated with this EO, rich in estragole, cell viability was augmented while ROS generation was modulated by increasing CAT and SOD activities; MDA levels were also reduced compared to cells which were treated only with insecticide [122]. Although these results show that fennel EO has antioxidant activity and reduces DNA damage, it could increase the viability of a cancerous cell line, even if not reported by the authors.

The essential oil from *Myrica rubra* Siebold & Zucc. leaves has been showed mild antioxidant activity in a non-cancerous cell line from a primary culture of rat hepatocytes, however, it demonstrated a strong pro-oxidative effect on Caco-2 cancer cells due to increased production of ROS [147]. Furthermore, this EO combined with doxorubicin improved its antiproliferative and pro-oxidant properties in cancer cells. The chemical composition of *M. rubra* EO presents β-caryophyllene (43%), α-humulene (22%), humulene epoxide I (8%), valencene (6%), epi-α-selinene (6%), γ-muurolene (3%), β-caryphyllene-oxide (3%) and transnerolidol (2%) [148]. As a side result, it is noteworthy that this EO showed a significant antiproliferative effect in several intestinal cancer cell lines [149]. Another study investigated the antioxidant capacity of carvacrol, thymol and their mixture (10:1) in Caco-2 cells by measuring ROS production. It was observed that carvacrol and the mixture at high concentrations induced oxidative stress, while at low concentrations showed protection against lipid peroxidation and protein oxidation induced by hydrogen peroxide [150].

EOs have shown different properties to redox conditions. On one hand, the antioxidant properties could reduce the damage associated with ROS production preventing the conversion of benign cells into cancer cells, as well as DNA damage, but on the other hand, a pro-oxidant condition could also be a strategy to attack cancerous tissues (Table 2). This antioxidant-prooxidant activity of sesquiterpenes has been already reported [151].

**Table 2:** In vitro Antioxidant activities of EOs and isolated compounds

Type of EO	Tested cell lines	Results	Refs.
*Allium roseum* bulb EO	HT-29 CaCo-2	↓Production of superoxide anion	[99]
Cinnamaldehyde Cinnamon bark extract	HCT-116 HT-29 FHC	↑Nrf2, ↑cellular glutathione ↓Oxidative stress	[142]
Thymol	HT-29	↑ Protection of the colonic epithelium against oxidative DNA damage	[145]
Geraniol	HT-29	↑Protection from DNA methylation damage	[145]
Carvacrol Thymol	Caco-2	↑Antioxidant properties against DNA strand breaks	[146]
Fennel (*Foeniculum vulgare*) EO	HCT-116	↑Cell viability, ↑Antioxidant properties ↑DNA protection	[122]
EO from *Myrica rubra* leaves	Caco-2	↑Selective pro-oxidative effect on cancer cells	[147]
Thymoquinone	DLD-1	↑Pro-oxidative effects ↑Apoptosis	[126]
Carvacrol	Caco-2	↑Pro-oxidative effects at high doses ↑Antioxidant effects at low concentrations	[150]

### Antimutagenic effects of EOs

As described in the previous section, some components of EOs can be considered potential antimutagenic compounds since they are capable of protecting DNA against ROS-induced toxicity. Thymol, geraniol and fennel EOs have demonstrated this potential antimutagenic effect due to their antioxidant properties [122, 145]. The essential oil from *Croton lechleri* Müll. Arg. stem bark showed a protective efficacy in Ames test against mutagenic heterocyclic amines such as 2-amino-3-methylimidazo-[4,5-f]quinoline and 2-amino-3,4- dimethylimidazo-[4,5-f]quinolone [98]. It might be due to the inhibition of the metabolic activation via P450 and the blocking of mutagen access to DNA. It also showed antiproliferative properties in the LoVo and HepG2 cell lines. This EO contains 76.93% of sesquiterpenes, being sesquicineole the major compound, and 18.89% of monoterpenes, being the limonene the major representative.

### Anti-inflammatory effects of EOs

Inflammation is initiated/mediated by oxidative stress, which induces cytokines (mainly TNF-α, IL-6 or IL-10) production in response to an external or pathophysiological agent [152]. Both ROS and cytokines may activate different lymphocytes to encounter inflammation [153, 154]. During the inflammatory process, other mediators, such as nitric oxide (NO), interleukin 1 beta (IL-1β), histamine or PAF may have a role in the harmful mechanism [23, 155, 156]. Chronic exposure to all these mediators may lead to increased cell proliferation, mutagenesis, oncogene activation, and angiogenesis [157, 158]. Usually, plant extracts have been proved as anti-inflammatory agents due to the presence of polyphenols, such as phenolic acids; however, EOs and monoterpenes have been scarcely tested as anti-inflammatory compounds in cancer conditions [159]. Chronic inflammation and its associated infections account for approximately 20% of cancer-related deaths [160–162]. Turmeric has been used as a medicinal herb for thousands of years for the treatment of various disorders. Although curcumin is the most studied active constituent of turmeric, accumulating evidence suggests that other components of turmeric have additional anti-inflammatory and anti-tumorigenic properties [163]. Some studies have shown that curcumin preparations containing turmerone and turmeric EOs revealed that anti-inflammatory cytokines including IL-10 and IL-11 as well as FOXP3 were upregulated in the colon. The combined treatment of curcumin and turmerone provides superior protection from dextran sodium sulfate-induced colitis than curcumin alone, highlighting the anti-inflammatory potential of turmeric [164].

Myrcene and α-pinene are monoterpenes found in the aerial parts (leaves, twigs and berries) of *Pistacia lentiscus* L. They have been well characterized for their antibacterial and anti-inflammatory properties. Nonetheless, poor information exists on their potential anticancer activity. An increasing number of studies has revealed that EOs from *P. lentiscus* L. trunk resin (namely mastic gum) which contains α-pipene, β-pipene, β-myrcine, linalool, trans-caryophyllene and camphene, may exert anticancer activity in several types of human neoplasia, including prostate and colon carcinomas as well as haematological malignancies [165–167]. Particularly, hexane and ethanolic extracts of mastic gum were shown to induce p53- and p21-independent G1-phase arrest followed by apoptosis in human colon cancer HCT-116 cells in vitro [168, 169].

Another research revealed a dose-dependent reduction of tumour cell viability induced by myrcene and α-pinene in Caco-2 cells. Intracellular ROS production slightly increased according to *P. lentiscus* EOs exposure, but it was one of the lowest ROS levels compared to other cell lines. Probably the reason was that the concentrations tested in this assay were too high (640 µg/mL) [170]. Previously, anti-inflammatory properties were reported for limonene, β-pinene and γ-terpinene, which reduced leukocyte migration to the damaged tissue and exhibited anti-inflammatory activity [171, 172].

*Thymus alternans* K. EO has also demonstrated anti-inflammatory properties and antiproliferative activity in HCT-15 and HCT-116 cells. Such effect was specifically due to nerolidol, the main volatile component of *T. alternans* [88]. This sesquiterpene was also responsible for the cytotoxic activity of *Comptonia peregrina* L. Coulter, a native plant from Canada used in traditional medicine against cancer, in the human colon adenocarcinoma cell line DLD-1 [88].

Table 3 summarizes the anti-inflammatory properties of the EOs tested on different colon cancer cell lines. Generally, monoterpenes and sesquiterpenes seem to be the most active compounds. These terpenes have not only demonstrated an anti-inflammatory effect, but also concomitant antiproliferative and antibacterial ones. As inflammation is related to oxidative stress, these results are well linked to those exposed in “Anti-inflammatory effects of EOs” section.

**Table 3:** Anti-inflammatory effects of EOs and isolated compounds

EO	Tissue or cells	Results	References
Turmerone	Mouse colon cells	Anti-inflammatory ↓IL-10,↓ IL-11, ↑FOXP3	[164]
EO from leaves, twigs and berries of *Pistacia lentiscus*	HCT-116	Antibacterial ↓Pro-inflammatory markers ↓NO, ↓PGE2, ↓TNF-α	[165–167]
Turmerone Tocotrienol	HT-29 HCT-116	↓Growth of colon cancer cells	[173]
Thymoquinone	COLO-205 HCT-116	↓Phosphorylation of p65 protein ↓NF-κB, ↑Apoptosis	[174]
Nerolidol	HCT-15 HCT-116 DLD-1	↓Pro-inflammatory cytokines ↓TNF-α, ↓IL-1β Antiproliferative	[88]

↑increase, ↓decrease

*TNF-α* Tumor necrosis factor alpha, *FOXP3* Forkhead box P3, *PGE2* Prostaglandin E2, *IL* Interleukin, *NO* Nitric oxide

## The synergistic anticancer effect of EOS associated with other bioactive compounds or conventional chemotherapy

The synergy between different compounds is a sought-after effect in the fight against cancer. Such effect of the essential polyphenolic compounds of curcumin, the EO of turmeric (ETO-Cur), and the tocotrienol-rich fraction (TRF) of the vitamin E isomers has been evaluated in HT-29 and HCT-116 cells. Indeed, the combined treatment, especially for ETO-Cur and TRF, showed synergistic potential in the 2 cell models. Similarly, in in vivo studies, HCT-116 cells xenograft in SCID mice were treated by ETO-Cur and TRF, which synergically acted to inhibit tumor volume. Moreover, even changes in microbial diversity were observed in xenograft mice treated with such EOs combination [173].

Various studies have reported that TQ could enhance anti-cancer potential when co-administered with several chemotherapeutic agents while reducing their toxic side effects [175]. Different compounds (menthol, trans-anethole) were investigated for the preparation of oil/water microemulsions for the delivery of methotrexate, and the ability of methotrexate-loaded microemulsions to inhibit cancer cell growth. Menthol and trans-anethole EOs led to cytotoxic microemulsions decreasing HeLa cells proliferation by MTT assay concluding that the oily component could play a role in the efficacy and safety of the microemulsions (for comparison α-tocopherol based-formulations showed opposite effects with increased cell proliferation) [176]. EO from the leaves of *M. rubra* also demonstrated an antiproliferative effect on Caco2 cells, with strong prooxidative effect. In addition, this EO was able to improve the antiproliferative and prooxidative activity of doxorubicin enhancing intracellular and nuclei accumulation, as previous described [147].

## Limitations, clinical challenges and future perspectives

A limitative aspect of using the EOs in CRC management is represented by variable concentrations of bioactive compounds. Many factors, including ecological and geographical conditions influence to the quality and quantity of EOs. The chemical composition and biological activities of EOs depend on various factors such as habitat, climatic conditions, seasonality, cultivation and harvesting and conservation practices, the type of soil, the different extraction procedures [177–180] as well as a substantial variability based on the part of the plant used for the extraction of EO. Altitude is an important factor affecting yield, composition, and biology of plant extracts. *Satureja thymbra* L. EO showed increased cytotoxic activity at an altitude of 661 m if compared to the same EO collected at 156 m above sea level. This result was obtained using the sulforhodamine B assay on HCT-116 colon cancer cells with an IC_50_ of 2.45 ± 0.21 μg/mL. In this case, the EO composition was prevailed by carvacrol (14.30%) [181]. Another important therapeutic limitation derives from the fact that it is not known exactly and completely the pharmacokinetic profiles of the essential oils, and due to their hydrophobic nature EOs can lead to poor bioavailability and pharmacodynamics issues.

Clinical challenges derived from the search for a natural therapy that includes EOs has led to attempts to find methods for administering the extracts. To overcome these issues, researchers explored the possibility to load nano-carriers with EOs, individually or combined with conventional chemotherapeutic agents. For example, A *Carum carvi* L. oil nanoemulsion system was tested on HT-29 cells, whereas it demonstrated a cytotoxic effect and apoptosis induction by increased gene expression of caspase-3. Besides, the authors suggested that the use of dietary supplements with nanoemulsions could potentially decrease the risk of cancer and that more research was needed to confirm this hypothesis [182]. This approach of drug delivery however has been poorly studied in CRC and EOs thus leaving the possibility to go down this route, at least in preclinical models. In order to improve the anticancer therapeutic potential and reduce the toxicity of bioactives compounds, new nanopharmaceutical forms for target transport such as nanoparticles, liposomes, nanocapsules, niosomes should be developed and researched [21, 22, 163, 183]. Alternatively, EOs can be combined with other more bioavailable compounds in order to harness their impact on human organism. For example, EOs can be used together with other plant natural derived products to search for an additive or a synergistic effect. Nonetheless, this combination scheme should be carefully evaluated, as sometime novel compounds put inside in a complex organism can lead also to antagonistic effects, an activity that need to be avoided. Despite remarkable anticancer activity of EOs in CRC and cancer in general, clinical trials that face the challenge of using such preparations in humans are still lacking. It is hoped that this gap will be rapidly filled in and that new works will explore the superb effects of EOs in CRC.

## Conclusion

Essential oils have been used in alternative medicine for a very long time, due to the healing properties that have been studied and demonstrated. Numerous experimental pharmacological studies have shown that they can inhibit the development of cancer and deserve to be used in prevention and even as adjuncts to classical chemotherapy. Therapeutic strategies to fight against CRC relay on surgery, radiotherapy, immunotherapy, and chemotherapeutic agents. EOs, defined as volatile chemical molecules from plants, can be potentially inserted in the last category of curative tools for the treatment of cancer. Though numerous advancements have been reported in surgery and chemotherapy in the last decades leading to progression of patient time survival and even in the increase of clinical conditions of affected patients, the death rate of CRC is still worrying healthcare system worldwide. This updated review showed scientific evidence on the potential anticancer effect of EOs in CRC. EOs can exhibit cytotoxic effects on living cells depending on type and concentration. In eukaryotic cells, EOs can act as prooxidants affecting inner cell membranes and organelles such as mitochondria. In some cases, changes in intracellular redox potential and mitochondrial dysfunction induced by EO can be associated with their capacity to exert antigenotoxic effects. EOs can interfere with several molecular targets in a pleiotropic fashion, but undeniably the cytotoxic activity of EOs is based on their individual components. In general, EOs (due to their lipophilic properties and low molecular weights) can cross cell membranes altering the phospholipid layers, increasing membrane fluidity, and leading to leakage of ions and/or other cytoplasmic content, thus inducing ATP reduction, alteration of pH gradient and loss of mitochondrial potential. In the light of these results, EOs can be a new therapeutic window and a potential adjuvant chemotherapy of CRC.

## Supplementary Information

**Additional file 1. **CRC preclinical models.

## Data Availability

Not Applicable.
