# Supplementary material for: Natural essential oils as a new therapeutic tool in colorectal cancer
Source: Cancer Cell Int. 2022 Dec 13;22:407. doi: 10.1186/s12935-022-02806-5 (PMC9749237; doi:10.1186/s12935-022-02806-5)
Supplement: Supplementary file 1 — Additional file 1. CRC preclinical models. [file 12935_2022_2806_MOESM1_ESM.docx]

**CRC cell models used for *in vitro* experimental studies**

Despite the enormous progress made in recent years, CRC and metastatic CRC remain the third cause of death among human cancer, underlining once again how it is necessary to continue and expand clinical and preclinical research in this serious disease. Nowadays, many models including cell cultures and animals are available to study CRC. They can mimic humans in different aspects of the disease and are consistently used by researchers all over the world, though each of them has its advantages and limits. Cell-based models including human and murine cancer cells have been established to support the study of the disease and drug efficacy in *in vitro* [1].

*Murine cell lines*

*In vitro* murine CRC models include cancer cell lines such as CT26, an N-nitroso-N-methylurethane-(NNMU)-induced undifferentiated colon carcinoma derived from BALB/c mice and available as single-cell suspensions, CRL-2638 and CRL-2639 from the American Type Culture Collection [1,2]). As well, MC 38 (Mouse Colon 38) cell line was isolated from a grade-III adenocarcinoma that was induced in a female C57BL/6 mouse using chemical agents [1,3]. Another used *in vitro* murine model is the DHD/K12/PROb (PROb) which consists of a colon carcinoma cell line derived from a chemically induced colon cancer in BDIX rats [4]. These cells have the advantage of being low passages, and thus less likely to possess additional mutations. However, murine cells are not well characterized (genetically and functionally) as compared to human cells and cannot completely resemble human cancer [1]. Recently, a robust three-dimensional ‘organoid’ *in vitro* culture system has been established to better mimic cancer and found to be suitable for studying cancer signaling pathways or for drug target identification. Murine CRC organoids derived from murine Apc-deficient intestinal adenomas can result from 4-OH-tamoxifen, KrasG12D/shRNA knockdown or CRISPR/Cas9 editing or culture of minced neonatal intestinal tissue within a collagen Wnt or R-spondin1-free gel. Their application in drug discovery can allow comparing side-by-side organoids and wild-type CRCs. However, their usefulness is still limited given their murine origin and the development of more interesting human CRC cell lines [1].

*Human cell lines*

*In vitro* CRC models include cancer cell lines from human sources and many characterized cell lines are available, virtually inexpensive for this purpose [5]. As these cells have a human origin, they can mimic or reproduce what happens in humans and many cell culture techniques enable the screening of numerous therapeutic molecules. Additionally, the use of *in vitro* CRC models also permits to characterize fundamental tumor processes, including cell migration and invasion, measurement of oxidative stress and cell viability, elucidation of signalling pathways, cell aggregation, cell cycle analysis and colonies formation as well as to screen for therapeutic compounds [5-7]. CRC cell line HCT-116 (S45 β-catenin mutant) and HT-29 cell line are examples of cell panels that provide an excellent *in vitro* model for dissecting the molecular mechanisms that control CRC biology. Both cell lines can also form spherical (3D) cultures and are used in tumor development studies in *in vitro* and *in vivo*. Certainly, they can better mimic tumor architecture and enrich cancer stem cell-like cells. However, the 2 cell lines are different in terms of morphology, phenotype, proliferative potential, distribution in the cell cycle phases and spherogenicity and the same concept applies to all CRC cells [8]. Other well-known cell lines include Caco-2, a human colon adenocarcinoma cell line derived from a tumor of the human colonic epithelium [9] and LS174T, a moderately well-differentiated primary colon adenocarcinoma that invaded the pericolonic fibro adipose tissue of a 47-year-old Caucasian woman [10]. These cells provide excellent *in vitro* models for studying mucin expression in CRC which helps understand the specific mechanisms responsible for mucin expression and regulation in colorectal tumorigenesis [11]. Besides, Kim et al., recently established 18 human CRC cell lines (SNU-NCC-376, SNU-NCC-61, SNU-2621B, SNU-2536C, SNU-2493, SNU-2465, SNU-2431, SNU-2423, SNU-2407, SNU-2373B, SNU-2359, SNU-2353B, SNU-2303, SNU-2297, SNU-2172, SNU-1983, SNU-1566) from 6 primary and 6 metastatic tumors tissues of Korean patients suffering from CRC [12]. The cells were studied and evaluated for their properties such as morphology, doubling time, and sensitivities to most of the anti-cancer drugs widely used for the cancer treatment; and they have widely studied the mutational profile of these strains. The authors eventually concluded that the 18 novel CRC cell cultures could be used to investigate gene alterations during CRC and thus serve as a good model for CRC drug development.

Overall, cell lines obtained from a patient affected by CRC might represent a successful tool for CRC studies. However, some negative aspects should be mentioned, such as the inability to reproduce the tridimensional interaction of the tumor cells within the peritumoral tissue (immune response, metastasis and angiogenesis), epigenetic alteration of tumor cells due to continuous maintenance in an artificial environment, the lack of intra-tumour heterogeneity and the loss of similarity with the original tumor due to multiple culture passages [5,13].

**CRC Animal models used for *in vivo* experimental studies**

Different animal models have been developed for CRC. This section summarizes the available literature for this experimental tool.

*3.2.1 Carcinogen-induced CRC mouse models*

The *in vivo* carcinogen CRC mouse model is the oldest one induced by the administration of specific compounds via oral gavage, intramuscular/intraperitoneal injection and/or *ad libitum* feeding [14]. Commonly used chemicals for induction of CRC in animals are dimethylhydrazine (DMH) and azoxymethane (AOM), which act by producing free radicals responsible for DNA oxidative damage of colon and liver cells. Other inducers responsible for colorectal tumors in mice are the N-methyl-N-nitroN-nitrosoguanidine (MNNG) and N-methyl-N-nitrosourea (MNU) which are direct alkylating agents that do not require metabolic activation [15-17]. The method is rapid and efficient, but its success depends on many factors, such as sex, age, genetic background, the immunological status of mice, their intestinal flora, and the diet. Other disadvantages of this method include its inability to produce invasive disease and metastasis, the necessity of a large number of animals to increase the incidence of tumor development, and the non-specificity of observed cancer which may not only be restricted to the colon but may be found in the whole gastrointestinal tract [18-20].

*Genetically engineered mouse model*

Globally, there is a great diversity of genetically modified animal models available for studying CRC [21,22]. Such models have indubitably contributed to the understanding of molecular processes of CRC initiation and progression. The genetically engineered mouse models (GEMM) have been developed and improved with the advent of genetic engineering tools, thanks to genomic information availability and mutation techniques [5,23]. Different genetic alterations in various classes of genes have been described. The most common modified genes are the growth-inhibiting cancer-suppressor genes including the tumor suppressors *APC* and *DCC* (Deleted in Colorectal Cancer), the oncogenes *KRAS*, *SRC* and *C-myc*, *TP53* and *MCC* (Mutated in Colorectal Cancer), genes that regulate apoptosis, the growth-promoting proto-oncogenes, and genes that regulate repair of damaged DNA viz. *hMSH2*, *hMSH6*, *hMLH1*, *CD44* genes and *COX-2*, in addition to *hPMS1* and *hPMS2* genes [20]. For example, mice with *APC* gene deletion can develop multiple adenomas throughout the gastrointestinal tract, but no tumor development in the colon. However, the use of the multiple intestinal neoplasias (Min) mouse model significantly develops colorectal adenomas. Also, the deletion of the *TP53* gene combined to a chemical agent was shown to increase the incidence rate of colon tumor development [20,24]. The GEMM also offers the possibility to study the implication of a specific gene in a particular signaling pathway and to know if a gene is involved in the pathogenesis of the disease [20]. However, the use of GEMM for preclinical studies is limited due to a lack of tumor heterogeneity since mice are inbred and therefore show insufficient genetic variability [23]. On one hand, as the tumors are developed through the modification of the same gene, the number of involved tumor pathways is limited. Furthermore, the short lifespan of the mouse does not allow sequential tumor progress up to the metastasis stage as compared to human disease [25,26]. This suggests that the GEMM model is effective to study the early stage of the disease and additional or alternative models are required for a better understanding of later stages of the disease.

*In vivo transplant models*

There are different transplant models available for CRC and this paragraph reports the techniques and models used in preclinical research.

Cell line xenograft

Xenograft models have been used to bypass the limitations of cell culture models. This method consists to implant graft or colorectal cancer cells from either human or murine (allograph) tumors into an immunosuppressed mouse (i.e. severe combined immunodeficient mice - SCID) thereby resulting in the growth of tumor at the injection site [5]. This model is widely used by researchers to induce CRC in mice for rapid carcinogenesis studies, response to therapeutics and target validation studies, but as for other methods, significant restrictions have been reported. Indeed, the role of the immune system in tumorigenesis cannot be well studied. As well, the environmental conditions in recipient mice injected into the subcutaneous space typically differ from that of the intestine or colon where the CRC is derived. This model beers low metastatic capability which hinders the good reproducibility of the disease [1,23].

Orthotopic model

The method is based on the direct implantation of the tumor inside the organ of interest (colon in case of CRC) in immunodeficient recipient mice. It is an alternative to subcutaneous implantation, that was developed to achieve a more reproducible liver metastasis in the late stages of CRC [5]. Though this approach results in a suit replication of the human disease with high reliability, the model requires the need of surgical skills and is subjected to some risks of incorrect parietal injection of tumor cells, low viability of tumor cells and host reaction to the cells may represent possible drawbacks [27-30].

Patient-derived xenograft

Patients’ derived xenografts (PDX) are another suitable approach to better simulate human tumor biology allowing for natural cancer progression. The method consists of the implantation of a patient’s tumor tissue into immunocompromised mice [1]. The PDX models allow strong preservation of the tumoral structure microscopically, genetically, functionally [13,31] and the development of personalized cancer therapy [32]. However, the model also presents some significant drawbacks, such as the huge cost of the process, the high length of screening therapies, and the replacement of the mice stroma with the human cells after three weeks and the fact that samples usually originated from highly advanced CRC patients [1,33].

**Metastatic model**

The final stage of CRC is the metastatic phase which is responsible for the majority of death, thereby requiring a suitable animal model that consistently mimics cancer dissemination as in humans [1,34]. The main target organ for CRC metastasis is the liver and the method used for the study of the disease progression of CRC is the direct inoculation of tumor cells into the liver parenchyma, also called intrahepatic tumor implantation [17,35,36]. Alternative methods for metastasis induction also include the injection of tumor cells into the portal vein through the spleen via the laparotomy process in immunodeficient mice because of reproducing the vascular spread of the disease [17] or the orthotopic transplantation approach which shows low success rate (3.3%) after a long transplantation period [37].

Numerous progress has been made in the improvement of models that better understand and manage the CRC disease from the early to the final phase. Certainly, each experimental technique has its advantages and limitations that should be considered whenever a particular result is wanted. In addition, it is to be kept in mind that these methods are frequently complementary and could be used together to implement specific effects or outcomes.

**References**

1. Golovko, D.; Kedrin, D.; Yilmaz Ö, H.; Roper, J. Colorectal cancer models for novel drug discovery. *Expert opinion on drug discovery* **2015**, *10*, 1217-1229, doi:10.1517/17460441.2015.1079618.

2. Kishimoto, H.; Momiyama, M.; Aki, R.; Kimura, H.; Suetsugu, A.; Bouvet, M.; Fujiwara, T.; Hoffman, R.M. Development of a clinically-precise mouse model of rectal cancer. *PloS one* **2013**, *8*, e79453, doi:10.1371/journal.pone.0079453.

3. Efremova, M.; Rieder, D.; Klepsch, V.; Charoentong, P.; Finotello, F.; Hackl, H.; Hermann-Kleiter, N.; Löwer, M.; Baier, G.; Krogsdam, A., et al. Targeting immune checkpoints potentiates immunoediting and changes the dynamics of tumor evolution. *Nature communications* **2018**, *9*, 32, doi:10.1038/s41467-017-02424-0.

4. Pierrefite-Carle, V.; Baqué, P.; Gavelli, A.; Brossette, N.; Benchimol, D.; Bourgeon, A.; Saint Paul, M.C.; Staccini, P.; Rossi, B. Subcutaneous or intrahepatic injection of suicide gene modified tumour cells induces a systemic antitumour response in a metastatic model of colon carcinoma in rats. *Gut* **2002**, *50*, 387-391, doi:10.1136/gut.50.3.387.

5. Young, M.; Ordonez, L.; Clarke, A.R. What are the best routes to effectively model human colorectal cancer? *Molecular oncology* **2013**, *7*, 178-189, doi:10.1016/j.molonc.2013.02.006.

6. Bradshaw-Pierce, E.L.; Pitts, T.M.; Tan, A.C.; McPhillips, K.; West, M.; Gustafson, D.L.; Halsey, C.; Nguyen, L.; Lee, N.V.; Kan, J.L., et al. Tumor P-Glycoprotein Correlates with Efficacy of PF-3758309 in in vitro and in vivo Models of Colorectal Cancer. *Frontiers in pharmacology* **2013**, *4*, 22, doi:10.3389/fphar.2013.00022.

7. Devarasetty, M.; Dominijanni, A.; Herberg, S.; Shelkey, E.; Skardal, A.; Soker, S. Simulating the human colorectal cancer microenvironment in 3D tumor-stroma co-cultures in vitro and in vivo. *Scientific reports* **2020**, *10*, 9832, doi:10.1038/s41598-020-66785-1.

8. Shaheen, S.; Ahmed, M.; Lorenzi, F.; Nateri, A.S. Spheroid-Formation (Colonosphere) Assay for in Vitro Assessment and Expansion of Stem Cells in Colon Cancer. *Stem cell reviews and reports* **2016**, *12*, 492-499, doi:10.1007/s12015-016-9664-6.

9. Biazik, J.M.; Jahn, K.A.; Su, Y.; Wu, Y.N.; Braet, F. Unlocking the ultrastructure of colorectal cancer cells in vitro using selective staining. *World journal of gastroenterology* **2010**, *16*, 2743-2753, doi:10.3748/wjg.v16.i22.2743.

10. Tom, B.H.; Rutzky, L.P.; Jakstys, M.M.; Oyasu, R.; Kaye, C.I.; Kahan, B.D. Human colonic adenocarcinoma cells. I. Establishment and description of a new line. *In vitro* **1976**, *12*, 180-191, doi:10.1007/bf02796440.

11. Bu, X.D.; Li, N.; Tian, X.Q.; Huang, P.L. Caco-2 and LS174T cell lines provide different models for studying mucin expression in colon cancer. *Tissue & cell* **2011**, *43*, 201-206, doi:10.1016/j.tice.2011.03.002.

12. Kim, S.C.; Kim, H.S.; Kim, J.H.; Jeong, N.; Shin, Y.K.; Kim, M.J.; Park, J.W.; Jeong, S.Y.; Ku, J.L. Establishment and characterization of 18 human colorectal cancer cell lines. *Scientific reports* **2020**, *10*, 6801, doi:10.1038/s41598-020-63812-z.

13. Brown, K.M.; Xue, A.; Mittal, A.; Samra, J.S.; Smith, R.; Hugh, T.J. Patient-derived xenograft models of colorectal cancer in pre-clinical research: a systematic review. *Oncotarget* **2016**, *7*, 66212-66225, doi:10.18632/oncotarget.11184.

14. Bürtin, F.; Mullins, C.S.; Linnebacher, M. Mouse models of colorectal cancer: Past, present and future perspectives. *World journal of gastroenterology* **2020**, *26*, 1394-1426, doi:10.3748/wjg.v26.i13.1394.

15. Narisawa, T.; Magadia, N.E.; Weisburger, J.H.; Wynder, E.L. Promoting effect of bile acids on colon carcinogenesis after intrarectal instillation of N-methyl-N'-nitro-N-nitrosoguanidine in rats. *Journal of the National Cancer Institute* **1974**, *53*, 1093-1097, doi:10.1093/jnci/53.4.1093.

16. Narisawa, T.; Weisburger, J.H. Colon cancer induction in mice by intrarectal instillation of N-methylnitosorurea (38498). *Proceedings of the Society for Experimental Biology and Medicine. Society for Experimental Biology and Medicine (New York, N.Y.)* **1975**, *148*, 166-169, doi:10.3181/00379727-148-38498.

17. Tong, Y.; Yang, W.; Koeffler, H.P. Mouse models of colorectal cancer. *Chinese journal of cancer* **2011**, *30*, 450-462, doi:10.5732/cjc.011.10041.

18. Nordlinger, B.; Panis, Y.; Puts, J.P.; Herve, J.P.; Delelo, R.; Ballet, F. Experimental model of colon cancer: recurrences after surgery alone or associated with intraperitoneal 5-fluorouracil chemotherapy. *Diseases of the colon and rectum* **1991**, *34*, 658-663, doi:10.1007/bf02050346.

19. Matsumoto, K.; Iwase, T.; Hirono, I.; Nishida, Y.; Iwahori, Y.; Hori, T.; Asamoto, M.; Takasuka, N.; Kim, D.J.; Ushijima, T., et al. Demonstration of ras and p53 gene mutations in carcinomas in the forestomach and intestine and soft tissue sarcomas induced by N-methyl-N-nitrosourea in the rat. *Japanese journal of cancer research : Gann* **1997**, *88*, 129-136, doi:10.1111/j.1349-7006.1997.tb00357.x.

20. Heijstek, M.W.; Kranenburg, O.; Borel Rinkes, I.H. Mouse models of colorectal cancer and liver metastases. *Digestive surgery* **2005**, *22*, 16-25, doi:10.1159/000085342.

21. de Castro Cotti, G.; de Souza Santos, F.; Sebastianes, F.; Habr-Gama, A.; Seid, V.E.; de Martino, R. Genética do câncer colorretal. *Revista de Medicina* **2000**, *79*, 65-72.

22. DE-Souza, A.; Costa-Casagrande, T.A. ANIMAL MODELS FOR COLORECTAL CANCER. *Arquivos brasileiros de cirurgia digestiva : ABCD = Brazilian archives of digestive surgery* **2018**, *31*, e1369, doi:10.1590/0102-672020180001e1369.

23. McIntyre, R.E.; van der Weyden, L.; Adams, D.J. Cancer gene discovery in the mouse. *Current opinion in genetics & development* **2012**, *22*, 14-20, doi:10.1016/j.gde.2011.12.003.

24. Donehower, L.A. The p53-deficient mouse: a model for basic and applied cancer studies. *Seminars in cancer biology* **1996**, *7*, 269-278, doi:10.1006/scbi.1996.0035.

25. Chen, X.; Halberg, R.B.; Burch, R.P.; Dove, W.F. Intestinal adenomagenesis involves core molecular signatures of the epithelial-mesenchymal transition. *Journal of molecular histology* **2008**, *39*, 283-294, doi:10.1007/s10735-008-9164-3.

26. Halberg, R.B.; Waggoner, J.; Rasmussen, K.; White, A.; Clipson, L.; Prunuske, A.J.; Bacher, J.W.; Sullivan, R.; Washington, M.K.; Pitot, H.C., et al. Long-lived Min mice develop advanced intestinal cancers through a genetically conservative pathway. *Cancer research* **2009**, *69*, 5768-5775, doi:10.1158/0008-5472.can-09-0446.

27. Boni, L.; Benevento, A.; Dionigi, G.; Rovera, F.; Diurni, M.; Dionigi, R. Injection of colorectal cancer cells in mesenteric and antimesenteric sides of the colon results in different patterns of metastatic diffusion: an experimental study in rats. *World journal of surgical oncology* **2005**, *3*, 69, doi:10.1186/1477-7819-3-69.

28. Tseng, W.; Leong, X.; Engleman, E. Orthotopic mouse model of colorectal cancer. *Journal of visualized experiments : JoVE* **2007**, 10.3791/484, 484, doi:10.3791/484.

29. Liao, H.W.; Hung, M.C. Intracaecal Orthotopic Colorectal Cancer Xenograft Mouse Model. *Bio-protocol* **2017**, *7*, doi:10.21769/BioProtoc.2311.

30. Hite, N.; Klinger, A.; Hellmers, L.; Maresh, G.A.; Miller, P.E.; Zhang, X.; Li, L.; Margolin, D.A. An Optimal Orthotopic Mouse Model for Human Colorectal Cancer Primary Tumor Growth and Spontaneous Metastasis. *Diseases of the colon and rectum* **2018**, *61*, 698-705, doi:10.1097/dcr.0000000000001096.

31. Puig, I.; Chicote, I.; Tenbaum, S.P.; Arqués, O.; Herance, J.R.; Gispert, J.D.; Jimenez, J.; Landolfi, S.; Caci, K.; Allende, H., et al. A personalized preclinical model to evaluate the metastatic potential of patient-derived colon cancer initiating cells. *Clinical cancer research : an official journal of the American Association for Cancer Research* **2013**, *19*, 6787-6801, doi:10.1158/1078-0432.ccr-12-1740.

32. Hidalgo, M.; Bruckheimer, E.; Rajeshkumar, N.V.; Garrido-Laguna, I.; De Oliveira, E.; Rubio-Viqueira, B.; Strawn, S.; Wick, M.J.; Martell, J.; Sidransky, D. A pilot clinical study of treatment guided by personalized tumorgrafts in patients with advanced cancer. *Molecular cancer therapeutics* **2011**, *10*, 1311-1316, doi:10.1158/1535-7163.mct-11-0233.

33. Dangles-Marie, V.; Pocard, M.; Richon, S.; Weiswald, L.B.; Assayag, F.; Saulnier, P.; Judde, J.G.; Janneau, J.L.; Auger, N.; Validire, P., et al. Establishment of human colon cancer cell lines from fresh tumors versus xenografts: comparison of success rate and cell line features. *Cancer research* **2007**, *67*, 398-407, doi:10.1158/0008-5472.can-06-0594.

34. Mitrut, P.; Docea, A.O.; Kamal, A.M.; Mitrut, R.; Calina, D.; Gofita, E.; Padureanu, V.; Gruia, C.; Streba, L. *Colorectal Cancer and Inflammatory Bowel Disease*; 2016; 10.5772/63408pp. 185-199.

35. Chen, H.J.; Yang, B.L.; Chen, Y.G.; Lin, Q.; Zhang, S.P.; Gu, Y.F. A GFP-labeled human colon cancer metastasis model featuring surgical orthotopic implantation. *Asian Pacific journal of cancer prevention : APJCP* **2012**, *13*, 4263-4266, doi:10.7314/apjcp.2012.13.9.4263.

36. Rashidi, B.; Gamagami, R.; Sasson, A.; Sun, F.X.; Geller, J.; Moossa, A.R.; Hoffman, R.M. An orthotopic mouse model of remetastasis of human colon cancer liver metastasis. *Clinical cancer research : an official journal of the American Association for Cancer Research* **2000**, *6*, 2556-2561.

37. Donigan, M.; Loh, B.D.; Norcross, L.S.; Li, S.; Williamson, P.R.; DeJesus, S.; Ferrara, A.; Gallagher, J.T.; Baker, C.H. A metastatic colon cancer model using nonoperative transanal rectal injection. *Surgical endoscopy* **2010**, *24*, 642-647, doi:10.1007/s00464-009-0650-9.
